# Impact of COVID-19 pandemic on interhospital transfer of patients with major trauma in Korea: a retrospective cohort study

**DOI:** 10.1186/s12873-024-00963-6

**Published:** 2024-04-03

**Authors:** Sung Hoon Cho, Woo Young Nho, Dong Eun Lee, Jae Yun Ahn, Joon-Woo Kim, Kyoung Hoon Lim, Hyun Wook Ryoo, Jong Kun Kim

**Affiliations:** 1 Trauma Center, Kyungpook National University Hospital, School of Medicine, Kyungpook National University, Daegu, Republic of Korea; 2 Department of Surgery, School of Medicine, Kyungpook National University, Daegu, Republic of Korea; 3 Department of Emergency Medicine, School of Medicine, Kyungpook National University, Daegu, Republic of Korea; 4 Department of Orthopaedic Surgery, School of Medicine, Kyungpook National University, Daegu, Republic of Korea

**Keywords:** Interhospital transfer, Major trauma, Trauma center, COVID-19

## Abstract

**Background:**

Interhospital transfer (IHT) is necessary for providing ultimate care in the current emergency care system, particularly for patients with severe trauma. However, studies on IHT during the pandemic were limited. Furthermore, evidence on the effects of the coronavirus disease 2019 (COVID-19) pandemic on IHT among patients with major trauma was lacking.

**Method:**

This retrospective cohort study was conducted in an urban trauma center (TC) of a tertiary academic affiliated hospital in Daegu, Korea. The COVID-19 period was defined as from February 1, 2020 to January 31, 2021, whereas the pre-COVID-19 period was defined as the same duration of preceding span. Clinical data collected in each period were compared. We hypothesized that the COVID-19 pandemic negatively impacted IHT.

**Results:**

A total of 2,100 individual patients were included for analysis. During the pandemic, the total number of IHTs decreased from 1,317 to 783 (− 40.5%). Patients were younger (median age, 63 [45–77] vs. 61[44–74] years, *p* = 0.038), and occupational injury was significantly higher during the pandemic (11.6% vs. 15.7%, *p* = 0.025). The trauma team activation (TTA) ratio was higher during the pandemic both on major trauma (57.3% vs. 69.6%, *p* = 0.006) and the total patient cohort (22.2% vs. 30.5%, *p* < 0.001). In the COVID-19 period, duration from incidence to the TC was longer (218 [158–480] vs. 263[180–674] minutes, *p* = 0.021), and secondary transfer was lower (2.5% vs. 0.0%, *p* = 0.025).

**Conclusion:**

We observed that the total number of IHTs to the TC was reduced during the COVID-19 pandemic. Overall, TTA was more frequent, particularly among patients with major trauma. Patients with severe injury experienced longer duration from incident to the TC and lesser secondary transfer from the TC during the COVID-19 pandemic.

## Introduction

The coronavirus disease 2019 (COVID-19) pandemic has significantly impacted the current healthcare system. Global healthcare providers have faced a historical crisis and have accumulated experiences and lessons during the pandemic. As a major part of critical care, the impact of the pandemic on trauma care has been investigated [1–3]. Overall road traffic volume reduction with social restriction, including lockdown or stay-at-home order, were observed and consequently resulted in a reduced number of motor vehicle accidents, emergency trauma surgeries, and trauma admissions [1–4]. In contrast, the number of road traffic accidents involving bicycles or electronic scooters, which were selected as an alternative method over existing public transportation to mitigate the spread of COVID-19, increased [5, 6]. Notably, the severity of trauma was reported similar regardless of the pandemic [5, 7]. Furthermore, some studies showed better outcomes such as total hospital length of stay (LOS) or intensive care unit (ICU) LOS for patients with severe injuries during that period [8].

To provide ultimate care in the current emergency care system, interhospital transfer (IHT) is necessary. Incomplete prehospital triage, lack of resources in a responsible trauma center (TC), or geographical distance from the scene possibly interferes with the timely direct transport to the definitive care facility. Particularly, IHT showed obvious benefits for patients with severe trauma [9, 10]. Nevertheless, during the pandemic, studies on IHT were limited to the practice within emergency departments or a particular surgical entity [11, 12]. Furthermore, evidence on the effects of the COVID-19 pandemic on IHT among patients with major trauma was lacking. Although, the potential reduction in the total IHT number during pandemic due to the aforementioned factors can be postulated, their practical influence has not been explored [1–4]. Therefore, this study aimed to analyze the impact of the COVID-19 pandemic on the IHT of patients with severe trauma in an urban TC in Korea. We hypothesized that the COVID-19 pandemic negatively impacted the practice of trauma care and their outcomes on IHT.

## Methods

### Study design

This retrospective cohort study was conducted in an urban TC of a tertiary academic affiliated hospital in Daegu, Korea. In 2012, the government of Korea assigned five trauma-focused facilities in the country, and this hospital was one of the five inaugural design centers. As the only TC in the metropolitan city with over 2 million citizens, their coverage is approximately 3 million individuals, including those from surrounding suburban areas. Data were retrieved from the institutional trauma registry, a part of the Korean Trauma Data Bank (KTDB), from February 1, 2019 to January 31, 2021. Eligible case was defined as one who was transferred from another medical facility. The study was approved by the Institutional Review Board (IRB) of Kyungpook National University Hospital (No. KNUH 2023-06-032), and individual consent for this retrospective study was waived by the IRB of Kyungpook National University Hospital.

### Data collection

Data on sex, age, nationality, type of injury, mechanism of injury, referring facility, mode of transport, total elapsed time from injury to arrival, vital signs and mentality score on arrival, blood product transfusion, trauma team activation (TTA), emergency surgery or intervention, Abbreviated Injury Scale score, Injury Severity Score (ISS), care in the ICU with LOS, and mortality were collected. We divided the total study duration into two parts according to the point of the COVID-19 outbreak in a metropolitan area in February 2020. Accordingly, the COVID-19 period was defined as from February 1, 2020 to January 31, 2021, whereas the pre-COVID-19 period was defined as the same duration of preceding span. Clinical data collected in each period were compared. Additionally, we performed a subgroup analysis in major trauma (ISS > 15) [13].

### Statistical analysis

Data were presented as means ± standard deviations or medians with interquartile ranges for continuous variables and as frequencies and percentages for categorical variables. The Shapiro–Wilk test was used to evaluate the normality of data. Student’s t-test or the Mann–Whitney U-test was used to compare continuous variables, and the chi-square test or Fisher exact test was used to compare categorical variables. All tests were two-tailed, and P-values < 0.05 were considered statistically significant. Data were analyzed using Statistical Package for the Social Sciences (version 27, IBM, Armonk, NY, USA) and Jamovi version 2.3.21(The Jamovi Project, Sydney, Australia).

## Results

**Fig. 1:**
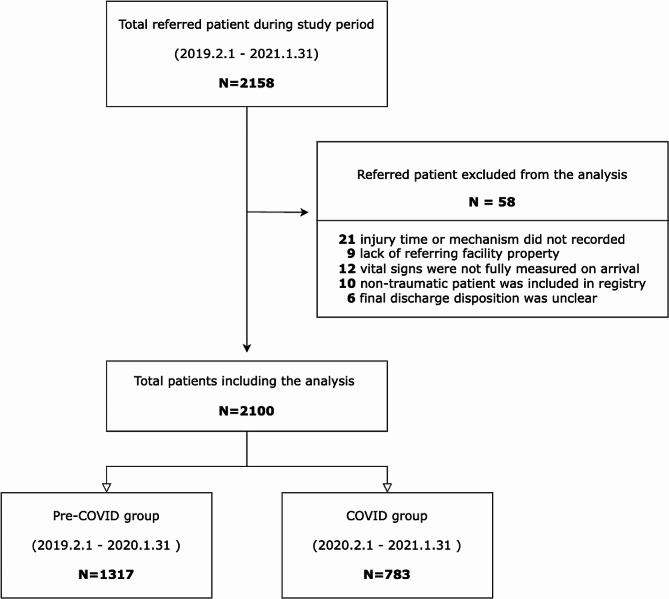
Flowchart of study participants selection

A total of 2,158 referred cases out of 6,234 total visitors were retrieved from the database. 58 were excluded due to missing values. Finally, a total of 2,100 patients were included for analysis (Fig. 1). During the pandemic, the total number of patients who underwent IHT decreased from 1,317 to 783 (− 40.5%). The proportion of IHT/total TC visitors was decre Patients during the COVID-19 pandemic were younger (mean age, 63 [45–77] vs. 61[44–74] years, *p* = 0.038). In both periods, blunt trauma and slip down were the most common injury mechanisms. Injury during occupational activity was significantly higher during the pandemic (11.6% vs. 15.7%, *p* = 0.025). Other demographic and general characteristics of enrolled patients are summarized in Table 1.

**Table 1:** Demographic characteristics and pre-transfer information according to the period

		Total (2019.2-2021.1)	Pre-COVID-19 (2019.2-2020.1)	COVID-19 (2020.2-2021.1)	*p-value*
Number of patients		2100	1317	783	
Age^a^					
		62 (45–76)	63 (45–77)	61 (44–74)	**0.038**
	Age ≥ 65	977 (46.5)	631 (47.9)	346 (44.2)	0.098
Sex					0.579
	Male	1317 (62.7)	820 (62.3)	497 (63.5)	
	Female	783 (37.3)	497 (37.7)	286 (36.5)	
Nationality					0.757
	Domestic	2070 (98.6)	1299 (98.6)	771 (98.5)	
	Foreign	30 (1.4)	18 (1.4)	12 (1.5)	
Injury type					0.684
	Blunt trauma	2044 (97.3)	1280 (97.2)	764 (97.6)	
	Penetrating	51 (2.4)	35 (2.7)	16 (2.0)	
	Burn	5 (0.3)	2 (0.1)	3 (0.4)	
Injury mechanism					0.153
	RTA	682 (32.5)	437 (33.1)	245 (31.3)	
	AVP	153 (7.3)	104 (7.9)	49 (6.2)	
	MVC	227 (10.8)	145 (11.0)	82 (10.5)	
	MCC	155 (7.4)	98 (7.4)	57 (7.3)	
	Bicycle	84 (4.0)	49 (3.7)	35 (4.5)	
	E-scooter	13 (0.6)	4 (0.3)	9 (1.1)	
	Industrial	44 (2.1)	31 (2.3)	13 (1.7)	
	Other NMV	6 (0.3)	6 (0.5)	0 (0.0)	
	Slip down	685 (32.6)	417 (31.7)	268 (34.2)	
	Fall	426 (20.3)	261 (19.9)	165 (21.0)	
	Struck	184 (8.8)	131 (9.9)	53 (6.8)	
	Other	123 (5.8)	71 (5.4)	52 (6.7)	
Occupational activity					**0.025**
	Yes	276 (13.2)	153 (11.6)	123 (15.7)	
	No	1378 (65.6)	876 (66.5)	502 (64.1)	
	Unknown	446 (21.2)	288 (21.9)	158 (20.2)	
Alcohol-related					0.458
	No drink (test confirmed) or not suspected	438 (20.9)	276 (21.0)	162 (20.7)	
	Suspected (not tested)	64 (3.0)	34 (2.6)	30 (3.8)	
	Drink (test confirmed)	106 (5.1)	67 (5.1)	39 (5.0)	
	Unknown	1492 (71.0)	940 (71.3)	552 (70.5)	
Referring from TC					0.941
	TC	117 (5.6)	73 (5.5)	44 (5.6)	
	Non-TC	1983 (94.4)	1244 (94.5)	739 (94.4)	
Referring ED level					0.789
	Level I ED	263 (12.5)	166 (12.6)	97 (12.4)	
	Level II ED	426 (20.4)	269 (20.4)	157 (20.3)	
	Level III ED	813 (38.9)	501 (38.0)	312 (40.3)	
	Non-ED	589 (28.2)	380 (28.9)	209 (26.0)	
	Military ED	1 (0.0)	1 (0.1)	0 (0.0)	
Transport mode					0.850
	Ground	2098 (99.9)	1315 (98.2)	783 (100.0)	
	Air	2 (0.1)	2 (0.2)	0 (0.0)	
Time from injury to TC (m)^a^		329 (182–1383)	326 (174–1369)	334 (199–1390)	0.214

Unless otherwise indicated, data are reported as number (percent)

^a^Data are reported as median (interquartile range)

RTA, road traffic accident; AVP, automobile vs. pedestrian; MVC, motor vehicle collision; MCC, motorcycle collision; NMV, non-motorized vehicle; TC, trauma center; ED, emergency department

Clinical factors and practical variables on TC are presented in Table 2. The proportion of TTA from overall incoming patients was higher during the pandemic with statistical significance (22.2% vs. 30.5%, *p* < 0.001). During the pandemic, the TTA ratio was higher at subgroup analysis on major trauma (57.3% vs. 69.6%, *p* = 0.006) as well as the total patient cohort. The total elapsed time from incidence to the TC was longer (218 [158–480] vs. 263 [180–674], *p* = 0.021), and secondary transfer without any intervention or admission was lower in the COVID-19 period (2.5% vs. 0.0%, *p* = 0.025) (Table 3).

**Table 2:** Analysis on clinical aspects and outcomes at the trauma center

		Total (*n* = 2100)	Pre-COVID-19 (*n* = 1317)	COVID-19 (*n* = 783)	*p-value*
Vital sign^a^					
	SBP (mmHg)	136 [115–156]	136 [116–156]	135 [113–155]	0.352
	DBP (mmHg)	80 [67–90]	79 [66–90]	80 [68–90]	0.250
	PR (/min)	85 [73–99]	86 [73–99]	85 [74–98]	0.943
	BT (^o^C)	36.8 [36.4–37.1]	36.8 (36.4–37.1]	36.9 [36.5–37.2]	0.677
	RR (/min)	18 [16–18]	18 [16–18]	18 [18–20]	0.074
	Saturation (%)	98 [96–99]	98 [96–98]	98 [96–99]	0.574
	SI	0.61 [0.49–0.76]	0.61 [0.49–0.76]	0.61 [0.50–0.75]	0.894
Cardiac arrest before arrival					0.509
		38 (1.8)	25 (1.9)	13 (1.7)	
GCS^a^					0.663
		15 [15–15]	15 [15–15]	15 [15–15]	
TTA					**< 0.001**
	Yes	532 (25.3)	293 (22.2)	239 (30.5)	
	No	1568 (74.7)	1024 (77.8)	544 (69.5)	
ISS^a^					0.546
		9 [4–14]	9 [4–14]	9 [4–14]	
	ISS > 15	508 (24.2)	314 (23.8)	194 (24.8)	0.119
Blood product (pRBC) transfusion					
	Required in first 4 h	263 (12.5)	160 (12.1)	103 (13.2)	0.501
	Amount(pint)^b^				
	<4 h	0.66 ± 2.78	0.66 ± 2.83	0.65 ± 2.69	
	4-24 h	1.18 ± 5.01	1.23 ± 5.16	1.09 ± 4.75	
TC stay (m)^a^					0.940
		288 [134–603]	282 [130–639]	306 [142–564]	
Direct OR/IR					0.350
		279 (13.3)	182 (13.8)	97 (12.4)	
ICU care					
	No of patient	695 (33.1)	432 (32.8)	263 (33.6)	0.711
	LOS (h)^b^	54.5 ± 151.9	55.0 ± 159.3	53.7 ± 138.6	0.853
2nd transfer					0.426
		384 (18.3)	234 (17.8)	150 (19.2)	
Mortality					
	Total	102 (4.9)	67 (5.1)	35 (4.5)	0.525
	Death < 24 h^†^	45 (2.1)	26 (2.0)	19 (2.4)	0.489
	< 48 h^†^	51 (2.4)	31 (2.4)	20 (2.6)	0.773
	< 7d^†^	70 (3.3)	43 (3.3)	27 (3.4)	0.821
	< 30d^†^	88 (4.2)	55 (4.2)	33 (4.2)	0.966

Unless otherwise indicated, data are reported as number (percent)

^a^Data are reported as median [interquartile range]

^b^Data are reported as mean ± standard deviation

^†^Numbers and percentage presented indicate the accumulated value on each designed period

SBP, systolic blood pressure; DBP, diastolic blood pressure; PR, pulse rate; RR, respiratory rate, BT, body temperature; SI, shock index; GCS, Glasgow coma scale; TTA, trauma team activation; ISS, injury severity scale; pRBC, packed red blood cell; TC, trauma center; OR, operation room; IR, intervention room; ICU, intensive care unit; LOS, length of stay

**Table 3:** Subgroup analysis among the major trauma group (ISS > 15)

		Total (*n* = 508)	Pre-COVID (*n* = 314)	COVID (*n* = 194)	*p-value*
Time from injury to TC (m) ^a^		235 [166–554]	218 [158–470]	263 [180–674]	**0.021**
SI^a^		0.67 [0.54–0.91]	0.67 [0.54–0.90]	0.67 [0.54–0.94]	0.972
GCS^a^		15 [11–15]	15 [11–15]	15 [11–15]	0.439
TTA		315 (62.0)	180 (57.3)	135 (69.6)	**0.006**
ISS^a^		22 [17–26]	22 [17–27]	21 [17–25]	0.256
Blood product (pRBC) transfusion					
Required in first 4 h		167 (32.9)	99 (31.5)	68 (351)	0.412
Amount (pint)^b^					
	< 4 h	2.05 ± 4.84	1.93 ± 4.82	2.11 ± 4.86	0.686
	4-24 h	3.40 ± 9.01	3.46 ± 9.16	3.29 ± 8.73	0.830
TC stay (m)^a^		151 [99–304]	142 [95–282]	157 [103–333]	0.153
Direct OR/IR		112 (22.0)	74 (23.6)	38 (19.6)	0.293
ICU care					
No of patient		414 (81.5)	255 (81.2)	159 (82.0)	0.833
LOS (h)^b^		165.6 ± 236.3	172.6 ± 254.6	154.2 ± 203.4	0.393
2nd transfer		8 (1.6)	8 (2.5)	0 (0.0)	**0.025**
Mortality					
	Total	77 (15.2)	50 (15.9)	27 (13.9)	0.540
	Death < 24 h^†^	36 (7.1)	20 (6.4)	16 (8.1)	0.423
	< 48 h^†^	42 (8.3)	25 (8.0)	17 (8.8)	0.750
	< 7d^†^	55 (10.8)	33 (10.5)	22 (11.3)	0.770
	< 30d^†^	65 (12.8)	39 (12.4)	26 (13.4)	0.748

Unless otherwise indicated, data are reported as number (percent)

^a^Data are reported as median [interquartile range]

^b^Data are reported as mean ± standard deviation

^†^Numbers and percentage presented indicate the accumulated value on each designed period

TC, trauma center; SI, shock index; GCS, Glasgow coma scale; TTA, trauma team activation; ISS, injury severity scale; pRBC, packed red blood cell; OR, operation room; IR, intervention room; ICU, intensive care unit; LOS, length of stay

## Discussion

In this study, we observed that the total number of IHTs to the TC decreased during the COVID-19 pandemic. Furthermore, patients were younger, and occupational injury was increased. Overall TTA was more frequent during the pandemic, particularly among patients with major trauma. Patients with severe injuries experienced longer duration from injury to the TC; however, no statistical difference was observed in time spent on the trauma bay or observation sector. Outcome variables, including blood product transfusion, direct emergency surgery or radiological intervention, ICU stay, and mortality, showed no significant difference. Notably, lesser secondary transfer was reported from the TC during the pandemic.

The major finding of our study is the reduced IHT during the pandemic. This is consistent with the result of a previous study, reporting reduced trauma transfer in Arizona [11]. The IHT is a multi-factorial process. Thus, clarifying specific components involved in the significantly reduced number of IHTs is complex. Notably, the significantly decreased number of patients who were injured and/or seeking medical service exclusively influenced the results. A multi-TC study in the United States showed decreased all-cause trauma admission [1], and a global study of 36 nations reported a decreased incidence of road traffic accidents with concomitant reduced number of patients with trauma during the pandemic [4]. Furthermore, a similar trend was shown in a domestic study [8]. Moreover, a temporary closure of regional EDs has affected those results. Geographically, Korea is closely located to the origin of the novel coronavirus. Further, in Korea, the city of Daegu is where the first epidemic occurred [14]. Several ED closures within the metropolitan area, including the institution where the study was conducted, were reported [15]. Thus, it may contribute to changes in the regional IHT pattern on both the referring and referred sides. Another consideration was focused on the mode of transport. In this study, the majority of transport was ground vehicles (99.9%). Notably, previous studies reported a shortage of ground transport utilization during the pandemic [16, 17]. We believed that the referring facility may experience failure on timely dispatch, which results in changes or waives their primary decision to move to the TC.

In this study, some demographic variables, including age and occupational relationship, showed statistical differences. Evidence of the relationship between the COVID-19 pandemic and those factors have been reported. A study conducted in the United Kingdom showed that more patients with trauma were younger during the pandemic [18]. Other studies conducted in the Netherlands and Japan reported a higher incidence of job-related injuries during the pandemic [19, 20]. We believed that our results were mainly derived from the changes in incidence during the pandemic. Although other factors may have impacted IHT, revealing the reasons in this study is difficult.

Our study highlights the higher TTA rate during the pandemic period in both the overall patient cohort and the group with severely injured patients. Trauma team organization is essential in the trauma care system. Each TC and team has its own TTA protocol, which can predict patients’ severity and achieve early response to care. TTA has great value for estimating major trauma, timely intervention, and consequently improving patient outcomes [21]. The higher TTA rate among IHTs during the pandemic indicates that more patients with severe injuries were referred to the TC. One considerable theory is traffic accidents due to harsh driving behaviors. During the pandemic, reduced road traffic volume influenced the decreased total number of injured patients. However, their severity increased because cars drove faster on empty lanes or roads with less traffic control [4]. High-speed collisions directly affected the increased number of fatalities on the road, and the increased use of cellphones or decreased compliance with seatbelt use contributed to the severity [22]. Moreover, TC visit following a high-speed motor accident is one of the inclusion criteria for TTA. Therefore, changes in traffic characteristics may affect the TTA rate by increased severity or more patients met the inclusion criteria. The tendency to make more IHT of patients with severe injuries to the TC is another possible factor. Surprisingly, the majority of outcome variables showed no difference in the pandemic despite higher TTA, and it might be the dedication of institutional trauma teams.

Longer elapsed time to the TC is another significant result from this study, and it may be affected by each of the time components. Delays may occur on the first responder dispatch, prehospital triage, selecting and moving to the initial facility, practice in the primary facility, and time spent on IHT. Some previous studies focused on adding extra time owing to several factors, including a shortage of prehospital or inhospital resources and performing and receiving the results of the COVID-19 testing during the pandemic [23]. Additional delays may occur during preparing an appropriate transport for IHT. In this study, we observed a median time from incidence to the TC arrival of 329 min. A study conducted in Denmark reported a time from injury to the TC of 255 min [24], and another study conducted in Canada showed 5.7 h from initial facility arrival to the TC [25]. Still, there is no standard recommendation on the optimal transfer time. However, longer dwelling time may contribute to adverse outcomes, and minimizing the time to definite care facilities was emphasized [26, 27].

The reduced secondary transfer without major intervention is an interesting finding in our study. The concept of futility includes death or hospice discharge, whereas the secondary overtriage (SO) indicates the unnecessary transfer who did not require surgery or radiological procedure [28, 29]. In this study, we noted a significant decrease in secondary transfer from the TC during the pandemic period. The exact cause of leaving without any intervention is unclear; however, we believed that patients were unsalvageable or did not require further intensive care. Given the factor consists of a part of futility or SO, we can estimate a decrease in futile transfers or SO to the TC in such period. This positive trend during the pandemic may have resulted from changes in the practice pattern or some involuntary reasons to candidacy the IHT more selectively.

This study had some limitations. First, the urban mono-TC–based retrospective design was an obvious limitation. Notably, IHT is more problematic among facilities in suburban or rural areas [30]. To generalize the results on this topic, certain participants should be included in future work. Second, despite the KTDB being one of the largest trauma databases in Korea, there was a significant lack of data, particularly on the referring side. Therefore, we were unable to collect key variables at initial facilities, including arrival time, exact information on the reason to decide IHT or limited resources, and time of leaving. In addition, because of the lack of a regional trauma registry, it was impossible to examine the proportion of IHTs, not actual numbers. Therefore, statistical demonstration was unavailable in the current study setting. Further efforts for establishing the regional transfer network or reinforcing the localized database are required.

## Conclusions

Our results showed that the total number of IHTs to the TC was reduced during the COVID-19 pandemic. Overall, TTA was more frequent, particularly among patients with major trauma. Patients with severe injuries experienced a longer duration from the incident to the TC and lesser secondary transfer from the TC during the COVID-19 pandemic.

## Data Availability

The datasets generated and/or analysed during the current study are not publicly available due information containing the privacy of the each patients, but are available from the corresponding author on reasonable request.

## Abbreviations

ICU: Intensive care unit
IHT: Interhospital transfer
ISS: Injury Severity Score
KTDB: Korean Trauma Data Bank
TC: Trauma center
TTA: Trauma team activation
SO: Secondary overtriage
